# Epidemiological Trends and Resistance Associated with *Stenotrophomonas maltophilia* Bacteremia: A 10-Year Retrospective Cohort Study in a Tertiary-Care Hospital in Hungary

**DOI:** 10.3390/diseases7020041

**Published:** 2019-05-31

**Authors:** Márió Gajdács, Edit Urbán

**Affiliations:** 1 Department of Pharmacodynamics and Biopharmacy, Faculty of Pharmacy, University of Szeged, 6720 Szeged, Hungary; 2 Institute of Clinical Microbiology, Faculty of Medicine, University of Szeged, 6725 Szeged, Hungary; tidenabru@freemail.hu

**Keywords:** *Stenotrophomonas maltophilia*, bacteremia, bloodstream infection, SMX/TMP, sumetrolim-resistance, retrospective, literature review

## Abstract

*Stenotrophomonas maltophilia* has been recognized as an emerging nosocomial pathogen in invasive infections of immunocompromised, severely debilitated patients with significant underlying illnesses. The first-choice drug in these infections is sulfamethoxazole-trimethoprim (SMX/TMP), and resistance to this antimicrobial is a daunting challenge for clinicians. The aim of this study was to assess the prevalence of *S. maltophilia* bacteremia and SMX/TMP-resistance levels at a tertiary-care university hospital. A total of 175 episodes of *S. maltophilia* bacteremia were identified (2008–2012: *n* = 82, 2013–2017: *n* = 93; *p* = 0.061), 52% of affected patients were 60 years of age, and had recent surgery, severe injuries or underlying conditions (malignant hematologic diseases and solid tumors) in their history. Sixteen percent of isolates were resistant to SMX/TMP (2008–2012: *n* = 13.8%, 2013–2017: *n* = 17.2%; *p* = 0.076), and out of the resistant strains, 32.7% were also resistant to levofloxacin and colistin. Our findings on the SMX/TMP-resistance were similar to global literature data.

## 1. Introduction

*Stenotrophomonas maltophilia* is a non-fermenting, oxidase-negative and catalase-positive aerobic Gram-negative bacillus [1,2]. *S. maltophilia* is the only human opportunistic pathogen of the *Stenotrophomonas* genus [2,3]. These bacteria are ubiquitous in the environment (frequently occurring in the soil and natural waters), and due to their positively charged cell wall surface and fimbriae, they can easily attach to and survive on various abiotic surfaces (e.g., irrigation fluids and tap water in the healthcare setting) [3,4,5,6]. *S. maltophilia* is also a biofilm-producer, which has a protective effect against drying-induced damage and hinders the functions of immune cells, inhibits the diffusion of antimicrobial drugs in vivo and allows for persistence in central venous catheters (CVCs) [3,4,5,6,7].

Before the 1970s, *S. maltophilia* was considered an unusual, opportunistic pathogen with a low rate of isolation and limited invasiveness because the pathogen must bypass the natural host defenses to cause infection [1,2,8,9]. However, owing to the advancements in medical interventions and pharmacological therapies (complex surgeries, cancer chemotherapy, immunosuppression for organ transplantation), in addition to the developments in more sensitive and precise identification methods in clinical microbiology, there have been more reports on *S. maltophilia* infections. Therefore, this pathogen has been recognized as an emerging nosocomial pathogen in invasive infections of immunocompromised, severely debilitated patients with significant underlying illnesses [7,10,11]. There are more and more reports of isolated *S. maltophilia* outbreaks in intensive care units (ICUs) and cancer wards [12,13]. The reported incidence of *S. maltophilia* nosocomial infections ranges between 7.1–37.7 cases/10,000 discharges, nevertheless, the incidence of these infections is directly proportionate to the number of patients at risk. Furthermore, since the 2000s, the relevance of this pathogen in community-acquired infections has also increased [14,15]. The most common manifestations of *S. maltophilia* infections are tracheobronchitis and bacteremia, however, their pathogenic role has also been described in meningitis, peritonitis [8,16,17,18], mastoiditis, ocular infections, infected wounds, skin and soft tissue infections, invasive bone and joint infections and infections of the genitourinary tract [8,9,19]. Many *S. maltophilia* infections are polymicrobial. The co-pathogens in these infections are usually other non-fermenting Gram-negative bacteria (*Pseudomonas aeruginosa*, *Acinetobacter* spp., *Burkholderia cepacia* complex) or *Staphylococcus aureus* [1,2,8,9]. *S. maltophilia* pulmonary infections are usually hospital-acquired and ventilator-associated (VAP). In patients with tumors of the hematopoietic and lymphoid tissues and severe immunosuppression (e.g., HIV-infection), a fulminant-course syndrome called fatal hemorrhagic pneumonia may occur [20,21]. *S. maltophilia* is also a well-known pathogen in cystic fibrosis patients; the infection rate correlates well with disease progression and loss of lung function, both in children and adults [22,23,24].

In *S. maltophilia* bacteremia, the source of the bacteria are usually the colonized/infected lungs, a CVC-infection or the gastrointestinal tract [25]. Risk factors include prolonged hospitalization, mechanical ventilation, admission to the intensive care unit (ICU), severe neutropenia and/or mucositis, various underlying diseases (hematological malignancy), cytotoxic chemotherapy or radiotherapy, corticosteroid therapy, recent surgical intervention or trauma, treatment with broad-spectrum antibiotics (most notably carbapenems), total parenteral nutrition (TPN), and previously identified *S. maltophilia* colonization [8,18,26,27]. *S. maltophilia* is a significant factor in morbidity and mortality, with the associated mortality of bacteremia thought to be around 20–70% [19,28]. The risk of relapse after therapy of catheter-associated bacteremia is very high, therefore the removal of the catheter and surgical debridement is also recommended in addition to anti-infective therapy [8,18,26,27].

From a clinical perspective, the management of *S. maltophilia* infections may be quite difficult [26]. This microorganism is intrinsically resistant to several classes of antibiotics, most notably aminoglycosides (LPS changes and an acetyl-transferase enzyme) and most of the β-lactam antibiotics (specifically carbapenems, owing to the production of a penicillinase (L1) and a cephalosporinase (L2) enzyme), due to various resistance mechanisms (e.g., efflux pumps) [1,29,30]. Thus, broad-spectrum antibiotic therapy may select for *S. maltophilia* in an in vivo environment, over other susceptible pathogens [8,18,26,27]. The therapy of choice in these infections is sulfamethoxazole/trimetoprim (co-trimoxazole; SMX/TMP), administered in 3–4 divided doses of 15 mg/kg/day [31,32,33]. Resistance to SMX/TMP is mediated by modifications in the target genes *sul1* and *sul2* [34,35]. Resistance rates in the Western Hemisphere range between 2–10%, however, some outliers with higher resistance levels in Europe (Spain: 27%), and in other parts of the globe (Turkey: 10–15%, Taiwan: up to 25%, China 30–48%) have been reported [29,34,35]. In patients with malignant neoplasms, ICU patients and cystic fibrosis (CF) patients, resistance levels may be even higher (20–80%) [10]. Resistance, hypersensitivity or expected severe side effects related to SMX/TMP present a serious challenge to clinicians, as the evidence supporting several antimicrobial drugs in *S. maltophilia* infections is not yet clear [36]. Nonetheless, a recent meta-analysis has found that fluoroquinolones (a popular alternative to SMX/TMP) are equally effective in the therapy of these infections [37]. For this reason, *S. maltophilia* has been listed by the World Health Organization as one of the most concerning multidrug-resistant (MDR), nosocomial pathogens worldwide [38,39,40]. Other drugs that may be considered (if *S. maltophilia* has shown in vitro susceptibility) are tetracyclines (doxycycline, minocycline and tigecycline), fluoroquinolones (ciprofloxacin, levofloxacin and moxifloxacin), ticarcillin/clavulanate, ceftazidime, colistin and some other antibiotics such as moxalactam, rifampin and chloramphenicol [17,41]. In the empiric therapy of *S. maltophilia* infections (especially in immunocompromised patients), SMX/TMP is usually combined with levofloxacin or ticarcillin/clavulanate, until susceptibility results are available, or if coverage of additional pathogens is necessary [17,41].

The epidemiology of *S. maltophilia* infections varies greatly by the profile of the healthcare institution and the geographic region. Therefore, the assessment of local data is essential to evaluate trends over time and to reflect on the national situation compared to international data. The aim of this study was to assess and describe the prevalence of bacteremia caused by *S. maltophilia* at a tertiary-care hospital in Hungary retrospectively, during a 10-year study period (2008–2017), to assess the resistance trends associated with this pathogen and to compare our findings with results of other studies in Hungary and from the international literature.

## 2. Materials and Methods

### 2.1. Study Design

This retrospective cohort study was carried out using microbiological data collected from between January 2008 to December 2017 at the Albert Szent-Györgyi Clinical Center (ASCC), an academic primary- and tertiary-care teaching hospital in Szeged, Hungary. ASCC has a bed capacity of 1820-beds (1465 active and 355 chronic beds) and annually serves more than 400,000 patients in the Southern Great Plain of Hungary [42]. An electronic search (January 2008 till December 2017) of the MedBakter laboratory information system (LIS) records were conducted by two authors (M.G. and E.U.). Isolates were considered separate if they occurred more than 14 days apart or S. maltophilia isolates with different antibiotic susceptibilities were detected. Time-to-positivity (TTP) data corresponding to the positive blood culture bottles was also collected. Polymicrobial bacteremia was defined by the isolation of more than one organism in a single blood culture. In addition, patient data was also collected on patients who had at least one positive aerobic blood culture for *S. maltophilia*, which was limited to demographic characteristics (age, sex) and the indication for blood culture sample submission.

### 2.2. Sample Processing, Identification and Antimicrobial Susceptibility Testing (AST)

The processing of blood culture bottles arriving to the Institute of Clinical Microbiology was carried out according to guidelines in routine clinical bacteriology. Between 2008–2012, the BD Bactec (Beckton Dickinson, Franklin Lakes, NJ, USA) detection system was employed in our Institute, while from 2013 onwards, the BacT/ALERT 3D (bioMérieux, Marcy-l*’*Étoile, France) detection system was used. Blood culture bottles were incubated for 5 days (21 days, if endocarditis was suspected) in the abovementioned detection systems.

Between 2008–2012, presumptive phenotypic/biochemical methods and VITEK 2 ID (bioMérieux, Marcy-l*’*Étoile, France) were used for bacterial identification, while after 2013, this was complemented by matrix-assisted laser desorption/ionization time-of-flight mass spectrometry (MALDI-TOF MS; Bruker Daltonik Gmbh. Gr.). The methodology of sample preparation for MALDI-TOF MS measurements is described elsewhere [43]. Antimicrobial susceptibility testing (AST) for SMX/TMP was performed using the disk diffusion method, the interpretation of the results was based on EUCAST breakpoints (http://www.eucast.org; zone diameter > 16 mm, MIC < 4 mg/L) (Liofilchem, Abruzzo, Italy). Susceptibility-testing for other antibiotics was performed in the case of SMX/TMP resistance: non-species specific (NSS) breakpoints were applied in the case of tigecycline (TGC; S ≤ 0.25 mg/L, R > 0.5 mg/L) and levofloxacin (LEV; S ≤ 1 mg/L, R > 2 mg/L) using the E-test (Liofilchem, Abruzzo, Italy). In the case of colistin (COL; S ≤ 4 mg/L, R > 4 mg/L), a *Pseudomonas aeruginosa*-specific breakpoint was used and testing was performed using broth microdilution in cation-adjusted Mueller-Hinton broth [34,44]. *S. aureus* ATCC 29213, *Enterococcus faecalis* ATCC 29212, *Escherichia coli* ATCC 25922 and *P. aeruginosa* ATCC 27853 were used as quality control strains.

In the case of a SMX/TMP-resistant isolate, the in vitro susceptibility testing of additional agents (see Section 1) is recommended. In addition, other antimicrobials need to be considered in patients where SMX/TMP may not be used (e.g., sulfur allergy, severe renal impairment or where dosing may not be reliable). EUCAST currently only provides validated disk diffusion breakpoints for SMX/TMP (in addition to the NSS breakpoints; http://www.eucast.org), while the Clinical and Laboratory Standards Institute (CLSI) offers dilution testing breakpoints for SMX/TMP, ceftazidime, chloramphenicol, levofloxacin, minocycline and ticarcillin/clavulanate, in addition to disk diffusion testing breakpoints for SMX/TMP, levofloxacin and minocycline [45]. However, for some drugs, there is insufficient evidence to establish breakpoints, therefore the clinical microbiologist cannot interpret susceptibility results in the susceptible/resistant duality.

### 2.3. Statistical Analysis

Descriptive statistical analysis (including means or medians with ranges and percentages to characterize data) was performed using Microsoft Excel 2013 (Redmond, WA, Microsoft Corp.). Additional statistical analyses were performed with SPSS software version 24 (IBM SPSS Statistics for Windows 24.0, IBM Corp. Armonk, NY, USA), using the χ^2^-test and two-sample-test (isolation frequency and resistance trends). *p* values < 0.05 were considered statistically significant.

## 3. Results

A total of 175 episodes of *S. maltophilia* bacteremia were identified (17.5 ± 11.36/year, range: 8–33 isolates; highest in 2013, lowest in 2009) during the study period. The number of isolates between 2008–2012 was *n* = 82, while for 2013–2017, this number was *n* = 93. A considerable, but not significant increase (*p* = 0.061; two-sample t-test) was observed in the isolation frequency in the second part of the study period (2013–2017). The affected patients presented with a slight male dominance (57.71%); the age distribution of patients was the following: 8.6% 0–5 years, 8.7% 6–17 years, 5.9% 18–35 years, 24.8% 36–59 years and 52.0% of patients were 60 years of age or older.

Indications for blood culture sample submission, associated with *S. maltophilia* bacteremia included hematological malignancies (predominantly acute myeloid leukemia and multiple myeloma) and solid tumors (lung, stomach and colon cancer) (21.1%), cardiovascular illnesses (17.7%), recent trauma (including polytrauma and fractures of the skull and spine) (14.9%), burns (5.1%) or other reasons (9.0%). Septicemia or other infectious processes, including meningitis, pleuritis or pneumonia were reported in 16.5% and 15.7% of cases, respectively. The origin of positive blood culture isolates from the ASCC corresponds to the abovementioned indications. The largest amount originated from the various wards of the Department of Internal Medicine (39.1%), Intensive Care Units of different profiles (cardiology–hematology, surgery and traumatology) (33.7%), the Department of Surgery (13.0%), the Department of Pediatrics (9.8%) and the Department of Neurology (4.4%).

In 78.4% of relevant blood culture samples, *S. maltophilia* was the only isolated pathogen, whereas in 21.6%, more than one (in 14.2% two and in 3.7% of both three and four) different species could be isolated (Table 1). Gram-negative rods (species of *Enterobacteriaceae* and other non-fermenting Gram-negative bacteria in equal measure; 44.0%) and Gram-positive cocci (44.0%) were the most frequent species co-isolated. *P. aeruginosa* (in 10 cases) was the most frequent co-isolate. Coagulase-negative staphylococci (CoNS) were also prevalent (in 17 cases), however, these strains are presumably just skin contaminants, associated with inadequate care during the sampling procedure for the blood culture bottles.

The average time-to-positivity (TTP) for the blood culture samples was 24.26 ± 19.98 h (range: 3.84–189 h). No significant association was observed during the change in blood culture incubation systems (23.1 vs. 24.52 h; *p* < 0.05); however, there were relevant differences between the TTPs of monobacterial and polymicrobial isolates (29.43 vs. 22.38 h; *p* = 0.032).

Eighty-four percent (range: 72–98%) of *S. maltophilia* blood culture isolates were susceptible to SMX/TMP during the 10-year study period. This left 16.0% of isolates (2008–2012: 13.8%, 2013–2017: 17.2%; *p* = 0.076; two-sample t-test) resistant to the first-line drug, SMX/TMP. Out of the SMX-TMP-resistant *S. maltophilia* strains that were tested with other antimicrobial drugs, 58.2% of isolates (9.4% overall) were also resistant to tigecycline. Additionally, 32.7% of isolates (5.2% overall) were also resistant to levofloxacin and colistin, based on non-species-specific (NSS) breakpoints and the colistin-breakpoint of *P. aeruginosa*, respectively.

## 4. Discussion, Literature Review

This study represents a large series of *S. maltophilia* bacteraemia in Hungary, accounting for a broad patient population, reported from a general tertiary care hospital, together with the resistance levels of the isolates for SMX/TMP, in addition to a range of antibiotics for SMX/TMP resistant strains. Although the resistance level of *S. maltophilia* is well-characterized in the literature from respiratory infections and cystic fibrosis patients, there is a relatively small amount of studies regarding S. maltophilia bacteremia and associated resistance levels. The following section is dedicated to the review of the relevant literature concerning this topic (summarized in Table 2).

In the study of Ebara et al., the retrospective epidemiological characterization of two medical hospitals were performed: the corresponding susceptibility rates of these isolates were 87.5% for SMX/TMP and 75.5% for levofloxacin [46]. In a retrospective, single-center study in Japan, spanning eight years, Hotta *el al.* identified 54 cases of clinically relevant *S. maltophilia* bacteremia, with SMX/TMP resistance levels around 18.0% and 100.0% minocycline susceptibility [47]. In addition, this study found that *S. maltophilia* bacteremia was associated with a significantly higher mortality rate (*p* = 0.012), compared to other non-fermenting Gram-negative pathogens. In a similar study, Sumida et al. reported 30 cases of *S. maltophilia* bloodstream infections and also found a significantly higher mortality rate (*p* = 0.047; in this study, resistance rates were not reported) compared to other non-fermenting Gram-negative bacteria [48]. During a 13-year-long retrospective, single-center survey in Japan, Araoka et al. found that the overall mortality rate associated with *S. maltophilia* bacteremia was as high as 51.0% [49]. In a single-center study, aiming pediatric patients, Wu et al. reported SMX/TMP resistance levels of 9.0% during a 10-year period, with a crude mortality rate of 40.6% [50]. Caylan et al. published the results of a 4-year-long survey of a Turkish hospital, in which the susceptibility of 190 *S. maltophilia* strains (67.9% had a nosocomial origin; 36.5% presented as bacteremia) was evaluated [51]. Ninety-four percent of strains were susceptible to SMX/TMP, while this number was significantly lower for ticarcillin/clavulanate and ciprofloxacin (79.0% and 53.5%, respectively). In another study from Turkey, Gozel et al. reported the epidemiological results of *S. maltophilia* infections in a 7-year period: 49.3% of infections were primary bacteremia and resistance rates were 17.1% for SMX/TMP and 2.9% for levofloxacin [52]. Ubeda et al. studied the prevalence and antibiotic resistance levels of a Spanish university hospital in a 7-year period. In this study, 78.0% of S. maltophilia cases were of nosocomial origin, and one-third of these cases were polymicrobial bacteremia. Ninety percent of isolates were susceptible to SMX/TMP, while ticarcillin/clavulanate susceptibility was 77.0% and ciprofloxacin susceptibility was 60.0% [53]. In a study of the same length, Garazi et al. reported 102 patients with *S. maltophilia* bacteremia in two tertiary care centers in New York [54]. This paper highlighted the importance of careful evaluation of CVCs, as most of the patents had a central venous access device (94.1%). Nosocomial acquisition was the predominant form (77.5%) and resistance levels to SMX/TMP, levofloxacin and ticarcillin/clavulanic acid were 2.9%, 7.1% and 50.8%, respectively. Muder et al. evaluated *S. maltophilia* bacteremia in multiple hospitals in Pittsburg and found 91 episodes; the 14-day mortality of the patients was 21.0%, while SMX/TMP susceptibility was 94.0% [55]. In a Canadian study, Church et al. screened invasive S. maltophilia isolates: out of 90 patients, 69.0% had a bloodstream infection, and 17.0% of the isolates were resistant to SMX/TMP. The same study also tested the efficacy of antibiotic combinations in vitro, and found that SMX/TMP + colistin and SMX/TMP + minocycline were the most effective [56]. Friedmann et al. performed a retrospective characterization of bloodstream infections in a tertiary-care hospital in Melbourne, Australia: 80.0% of cases were of nosocomial origin and the susceptibility rates of the strains were SMX/TMP 80%, chloramphenicol 75.5% and ceftazidime 64.5% [57]. In a retrospective, single-center study from Korea, Cho et al. recorded *S. maltophilia* bloodstream infections: 31 cases were identified, with susceptibility levels of 88.9%, 40.7% and 44.0% for SMX/TMP, ticarcillin/clavulanic acid and levofloxacin, respectively [58]. A Greek 6-year-long, single-center study by Samonis et al. evaluated the susceptibility of invasive S. maltophilia isolates: infection-related mortality was 14.7%, while resistance levels were 14.7%, 8.8%, 14,7% and 17.6% for SMX/TMP, colistin, netilmicin and ciprofloxacin, respectively [59]. Wu et al. reported the results of the Taiwan Surveillance of Antimicrobial Resistance (TSAR; 1998–2008), which enrolled 38 hospitals, concluding that SMX/TMP (17.5% resistance) and minocycline (96.0% susceptibility) remained the two antimicrobials with the best in vitro activities against *S. maltophilia* [60]. Meyer et al. reported the results of the SARI (Surveillance of Antimicrobial Use and Antimicrobial Resistance in German Intensive Care Units) prospective unit- and laboratory-based surveillance system in Germany [61]. The study found that the resistance levels in *S. maltophilia* strains isolated from German ICU patients were the following: SMX/TMP 10.3%, ciprofloxacin: 35.8% and piperacillin/tazobactam 60.7%. Compared to our results in the Southern Great Plain of Hungary, Juhász et al. found somewhat differing susceptibility patterns in a 3-year-long, single-center survey in the Central Region of Hungary: the *in vitro* susceptibilities were 99.0% for SMX/TMP, 75.0% for levofloxacin, 12.0% for tigecycline and 9.0% for colistin [44]. The same study group characterized the susceptibilities of SMX/TMP-resistant isolates and the efficacy of drug combinations in the same region. They found that 83.3% of isolates carried the *sul1* gene and none of these strains were susceptible to colistin or tigecycline, while levofloxacin/moxifloxacin was effective in only 37% of cases. At the same time, the most effective combination in vitro was moxifloxacin and ceftazidime [34,62].

In several cases (both in the literature findings, and in our own experience), *S. maltophilia* was isolated with another significant pathogen. This may bring additional therapeutic challenges, especially if the mentioned co-pathogen is also resistant to several antibiotics (e.g., carbapenem-resistant *Pseudomonas* and *Acinetobacter*, extended-spectrum β-lactamase-producing *Enterobacteriaceae*, methicillin-resistant *S. aureus* [MRSA]) [63,64,65]. Although evidence of the benefits of combination therapy is limited and its precise role in clinical practice is uncertain, there is an increasing amount of literature regarding its efficacy (especially in cases of SMX/TMP-resistance). Combinations usually include 2–3 agents, in the following setup: SMX/TMP or fluoroquinolones plus ceftazidime or ticarcillin/clavulanate plus minocycline/tigecycline and/or aminoglycosides and/or colistin and/or rifampin and/or chloramphenicol [7,31]. If in vitro susceptibility to these drugs is observed, a synergy assay (using the E-test synergy method or the checkerboard microplate method) is recommended before the administration of these drugs to the patients [66,67]. The intravenous dosing of colistin (the desirable plasma concentration is around 2 mg/L) is difficult due to the pharmacokonetic/pharmacodynamic properties of the drug, its nephrotoxicity and the interpatient variability. The latter is especially challenging in patients with renal failure and severe underlying conditions [68]. Therefore, the use of inhalational/aerosolized colistin (especially if the source of the bacteremia is the lungs) is gaining increasing attention [69,70].

## 5. Conclusions

*S. maltophilia* bloodstream infections are thought to be infrequent, however, their relevance is increasing in the era of invasive surgical interventions and heavily immunosuppressed patients, due to cancer chemotherapy and transplantation. These infections are associated with high morbidity and mortality, especially in the abovementioned, vulnerable patient groups. There is no clear answer whether *S. maltophilia* bacteremia is a cause of death or a marker of poor prognosis in these patients (i.e., advanced malignancies, prolonged mechanical ventilation and/or ICU stay, and long courses of broad-spectrum antibiotics), and this remains a controversial topic. The aim of the present study was to highlight some of the features of these infections locally and compare our findings with international trends. In line with the literature, *S. maltophilia* bacteremia mainly affected post-operative patients, patients with severe injuries or underlying conditions (malignant hematologic diseases and solid tumors) and patients over 60 years of age. The ratio of SMX/TMP-resistant strains was similar to the global average (5–20%), but somewhat higher than the reports of other European countries. Some limitations of this study must be acknowledged. Firstly, due to the inability to access the medical records of the individual patients affected, the presence and nature of symptoms of the patients were unknown. For the same reason, the correlation between the presence/absence of all relevant risk factors and *S. maltophilia* bacteremia could not be assessed. There is a risk of selection/referral bias, as studies describing the prevalence of infectious diseases and resistance trends are mainly tertiary-care centers, which generally correspond to patients with more severe conditions or underlying illnesses, compared to community-based settings [71].

Due to the selection pressure of the more pronounced use of carbapenem antibiotics, the laboratories should expect a more frequent occurrence of SMX/TMP-resistant strains. Treatment of confirmed *S. maltophilia* infections should be started as soon as possible, as delays in the selection of appropriate therapy may contribute to an increase in mortality. The increasing prevalence of SMX/TMP-resistant isolates is a serious concern for clinicians. Institutions must establish therapeutic protocols for these cases, based on local resistance trends and international guidelines. In addition, the relevance of various combination therapies needs to be further evaluated in a controlled, clinical setting. The appropriate sampling technique and consultation between treating physicians and clinical microbiologists is essential for the adequate treatment of these patients.

## Figures and Tables

**Table 1:** Bacteria co-isolated with *S. maltophilia* in bacteremia, 2008–2017.

Co-Isolates in Relevant Blood Cultures	Frequency (n)
*P. aeruginosa*	10
*S. epidermidis*	7
*S. hominis*	7
*E. faecalis*	5
*E. coli*	4
*Candida albicans*	4
*Enterobacter cloacae*	3
*Klebsiella pneumoniae*	3
*S. aureus (including MRSA)*	3
*S. haemolyticus*	3
*E. faecium*	2
*K. oxytoca*	2
*Acinetobacter baumanii*	1
*A. jejunii*	1
*A. lwoffi*	1
*C. krusei*	1
*C. parapsilosis*	1
*Chryseobacterium indologenes*	1
*Corynebacterium spp.*	1
*E. asburiae*	1
*Leclercia adecarboxylata*	1
*Proteus mirabilis*	1
*Proteus monteilii*	1
*Serratia marcescens*	1
*Streptococcus dysgalactiae*	1

**Table 2:** Summary of the literature regarding susceptibility trends of *S. maltophilia* [34,46,47,48,49,50,51,52,53,54,55,56,57,58,59,60,61,62].

			Ratio of Susceptible Isolates						
First Author	Study Years	Country	SMX/TMP	CIP or LEV	TIC/CLAV	CEFTZ	MINO	COL	Other
Friedmann et al.	1988–1997	Australia	80.0%	-	-	64.5%	-	-	Chloraphenicol: 75.5%
Ubeda et al.	1990–1996	Spain	90.0%	60.0%	77.0%	62.0%	-	-	-
Muder et al.	1991–1994	USA (Pittsburg)	94.0%	-	-	-	-	-	-
Wu et al.	1993–2003	China	91.0%	-	-	-	-	-	-
Wu et al.	1998–2008	China	82.5%	-	-		96.0%	-	-
Church et al.	1999–2009	Canada	83.0%	-	-	-	-	-	-
Caylan et al.	2000–2003	Turkey	94.0%	79.0%	53.5%	-	-	-	-
Garazi et al.	2001–2007	USA (New York)	97.1%	92.9%	49.2%	53.0%	-	-	-
Meyer et al.	2003–2004	Germany	89.7%	64.2%	-	-	-	-	Piperacillin/tazobactam: 29.3%
Samonis et al.	2005–2010	Greece	85.3%	82.4%	-	-	-	91.2%	Netilmicin: 85.3%
Hotta et al.	2005–2013	Japan	82.0%	-	-	-	100.0%	-	-
Gokhan et al.	2006–2013	Turkey	82.9%	97.1%	-	-		-	-
Ebara et al.	2007–2013	Japan	87.5%	75.5%	-	-	-	-	-
Juhász et al.	2009–2011	Hungary	99.9%	75.0%	-	-		9.0%	Tigecycline: 12.0%
Cho et al.	2009–2014	Rep. of Korea	88.9%	44.0%	40.0%	-	-	-	-

## Abbreviations

AST	antimicrobial susceptibility testing
ASCC	Albert Szent-Györgyi Clinical Center
CLSI	Clinical and Laboratory Standards Institute
CF	cystic fibrosis
CoNS	coagulase-negative *Staphylococcus*
COL	colistin
CVC	central venous catheter
EUCAST	European Committee for Antimicrobial Susceptibility Testing
HAP	hospital-acquired pneumonia
HIV	human immunodeficiency virus
ID	identification
ICU	intensive care unit
LEV	levofloxacin
LPS	lipopolysaccharide
MALDI-TOF MS	matrix-assisted laser desorption-ionization time-of-flight mass spectrometry
MDR	multidrug-resistant
NSS	non species-specific breakpoints
SMX/TMP	sulfamethoxazole-trimetoprim
TGC	tigecycline
TPN	total parenteral nutrition
TTP	time-to-positivity
VAP	ventilator-associated pneumonia
